# Self‐assessment with pediatric inflammatory bowel disease endoscopy scoring tools: A quality improvement pilot study

**DOI:** 10.1002/jpr3.70002

**Published:** 2025-02-06

**Authors:** Alexandra S. Hudson, Ghassan Wahbeh, Dale Lee, Kendra L. Francis, David Suskind, Hengqi Betty Zheng

**Affiliations:** ^1^ Division of Pediatric Gastroenterology, Seattle Children's Hospital University of Washington Seattle Washington USA

**Keywords:** Crohn's disease, fellow, ulcerative colitis

## Abstract

Endoscopy is a fundamental component pediatric gastroenterology (GI), with one of its primary applications being the evaluation and management of patients with inflammatory bowel disease (IBD). However, there is variation in education and experience of gastroenterologists with pediatric IBD endoscopy, which can affect the quality of patient care. This quality improvement pilot study assessed familiarity and comfort with key endoscopic assessment tools (13 questions) pre‐ and post‐ an evidence‐based group presentation. Both fellow and attending pediatric gastroenterologists had low levels of comfort with endoscopic Crohn's disease scoring, IBD phenotype classification, postoperative IBD scoring, and IBD dysplasia screening. The review presentation significantly enhanced their comfort, with this effect persisting for several months among fellows. To improve the quality of care for IBD patients, it would be beneficial to periodically review endoscopic guidelines in continuing medical education for pediatric gastroenterologists providing IBD care.

## Abstract (highlights)

**What is Known:**

- Endoscopy is an integral part of a pediatric gastroenterologist's practice, with inflammatory bowel disease (IBD)(IBD) being one of the most common indications.
- There is significant education variation on pediatric IBD endoscopy, which can impact patient care.

**What is New:**

- Pediatric gastroenterology fellows and attendings have low levels of comfort with Crohn's disease scores, IBD phenotype classification, postoperative IBD scores, and IBD dysplasia screening endoscopy.
- An evidence‐based review presentation improved comfort for all, with sustained fellows’ improvement for several months despite no rereview.
- To improve IBD patient care, periodic review of endoscopic guidelines for pediatric gastroenterologists should be initiated.

## 1 INTRODUCTION

Endoscopy is a fundamental component of a pediatric gastroenterology (GI) practice. While mastering the technical skills is essential, the cognitive aspect of endoscopy is frequently overlooked. This includes both identifying and interpreting endoscopic findings, which are integrated into clinical decision‐making and patient care.^1^ This is particularly important in inflammatory bowel disease (IBD), which is one of the most common indications for pediatric endoscopy.^2^ Even if a pediatric gastroenterologist does not specialize in IBD within their own practice, they will likely perform endoscopy to rule out IBD, or follow‐up on IBD patients referred by their colleagues.

The International Pediatric Endoscopic Quality Improvement Network (PEnQuIN) has been developed to improve and promote high‐quality pediatric endoscopy.^3^, ^4^, ^5^ This initiative is imperative as training and exposure to pediatric endoscopy is significantly varied between centers and fellowships. Despite the availability of published guidelines, consensus papers, and standardized scoring systems for pediatric IBD endoscopy, it remains unclear how familiar pediatric GI fellows and physicians are with these resources and how frequently they utilize them.^5^, ^6^ The objective of this quality improvement pilot study was to assess pediatric GI fellows' and attendings' comfort with key pediatric IBD endoscopic assessment tools, and to evaluate the effect of a group review summarizing the evidence‐based literature. Familiarity of endoscopic landscapes leading to comfort in utilizing assessment tools was a key measure in this study, as this increases likelihood of using a tool in real‐world clinical practice.^5^, ^6^

## 2 METHODS

### 2.1 Procedure

All current Pediatric GI fellows and Pediatric GI attendings at Seattle Children's Hospital, a tertiary pediatric hospital with a Pediatric IBD center, were invited to participate. This study was exempt from the local Seattle Children's Hospital Institution Research Board (IRB). Fellows (first‐, second‐, and third‐years) had completed an average of 50 colonoscopies per fellow per year. All attendings perform endoscopy on pediatric patients with suspected and established IBD as a part of their regular practice. Approximately 1000 colonoscopies occur per year.

The educational review was delivered via a hybrid presentation (both in‐person and on video conference call) over 30 min. Thirty minutes was chosen to fit into the standard allotted time for Division Educational rounds (usually 45–60 min), and to allow time for discussion and questions. To maximize engagement and session completion, the review was fit into one session instead of running multiple sessions. It was presented to the GI fellows in February 2024 and to attendings in May 2024. Pre‐ and post‐questions were completed on paper or via electronic word document at the time of the presentation. Fellows also completed the postreview questions again in May 2024 (3 months later) without repeat review. This 3‐month time period was chosen to allow enough time for the fellows to participate in multiple endoscopy procedure days. This would allow for spaced repetition of their review knowledge, which has been shown to significantly improve medical education knowledge retention.^7^

### 2.2 Review

Presentation slides were made by a pediatric advanced IBD fellow (A.H.) using the most up‐to‐date published guidelines, consensus, and position papers on pediatric IBD endoscopy. This included searching for publications by the North American Society of Pediatric Gastroenterology, Hepatology, and Nutrition (NASPGHAN),^3^, ^4^ European Society of Pediatric Gastroenterology, Hepatology, and Nutrition (ESPGHAN),^3^, ^4^, ^8^ European Crohn's and Colitis Organization (ECCO),^9^ and American Gastroenterology Association (AGA).^10^ The most frequently used standardized scoring systems and classifications in the pediatric IBD literature were included in the review: the Paris classification for Crohn's disease (CD) and ulcerative colitis (UC),^11^ the Simple Endoscopic Score for Crohn's Disease (SES‐CD),^12^ Mayo endoscopic sub‐score,^13^ Rutgeerts postoperative score,^14^ and Boston Bowel Preparation Scale.^15^ Anatomy and endoscopic landmarks of esophagogastroduodenoscopy, colonoscopy, ileoscopy, and pouchoscopy were included as well.

### 2.3 Questions

Questions were designed to be completed in less than 5 min immediately before and after the review session. Thirteen questions were created based on the above literature. All questions asked if the individual was comfortable with or aware of that particular pediatric IBD endoscopy topic (Supporting Information: Appendix A). Individuals scored each question on a Likert scale of 1–5 (1 = strongly disagree, 2 = disagree, 3 = neutral, 4 = agree, 5 = strongly agree). This was chosen as a simple way to measure general familiarity with a subject.

### 2.4 Statistical analyses

Descriptive statistics included median and 25th–75th interquartile ranges (IQR) for continuous variables, with frequency and percentage for categorical variables. Mann–Whitney *U* test compared responses between attendings and fellows. Wilcoxon signed‐rank test compared individuals' paired pre‐ and postreview questions, as well as fellows' paired post‐ and 3 month postreview questions. Spearman's rho correlated years of experience (years since pediatric GI fellowship start date) to pre‐ and post‐review total scores. All analyses were done in SPSS Version 20 (IBM® SPSS^©^ Statistics, 1 New Orchard Road Armonk, New York), and a *p*‐value less than 0.05 was considered statistically significant.

### 2.5 Ethics statement

After discussion with our local Seattle Children's Hospital IRB, this study was deemed a QI project and exempt from IRB. All participants in this study provided informed consent before initiation.

## 3 RESULTS

### 3.1 Participants

Fourteen individuals (*n* = 5/6 fellows, (83%), *n* = 9/19 attendings (47%)) participated. Fellows were from all years of training (*n* = 2 first‐year, *n* = 2 s‐year, and *n* = 1 third‐year). Attendings had a median of 11 years since fellowship graduation (IQR 7.5–16.5, range 5–24). In addition to all attendings practicing general pediatric GI and performing pediatric endoscopy, main clinical interests included IBD, hepatology, motility, and intestinal rehabilitation.

### 3.2 Overall comfort

The topics with the lowest levels of comfort (median score <3, indicating strongly disagree (=1) or disagree (=2)) shared among all fellows and attendings was the Rutgeerts postoperative score (median 2, IQR 1–2.3), IBD dysplasia screening endoscopic techniques (median 2, IQR 1–3), and the Paris classification (median 2, IQR 2–4) (Table 1). Pouchoscopy (median 2, IQR 2–4) and SES‐CD (median 2.5, IQR 2–3) were also topics of low comfort for all. The fellows identified four more topics of low comfort compared to the attendings (Table 1).

**Table 1:** Top pediatric inflammatory bowel disease (IBD) endoscopy topics where the median response regarding topic comfort/awareness was “strongly disagree” or “disagree.”

Overall (*n* = 14)	Fellows (*n* = 5)	Attendings (*n* = 9)
1. Rutgeert postoperative endoscopic score^a^	1. Rutgeert postoperative endoscopic score	1. Rutgeert postoperative endoscopic score^c^
2. IBD dysplasia screening endoscopic techniques^a^	2. IBD dysplasia screening endoscopic techniques^b^	2. IBD dysplasia screening endoscopic techniques^c^
3. Paris classification for CD and UC^a^	3. Paris classification for CD and UC^b^	3. Paris classification for CD and UC^c^
4. Pouchoscopy landmarks and findings^a^	4. Pouchoscopy landmarks and findings^b^	
5. SES‐CD endoscopic score	5. SES‐CD endoscopic score^b^	
	6. Ileoscopy landmarks and findings^b^	
	7. “Normal” non‐IBD endoscopic findings^b^	

*Note*: ^a,b,c^Same median score.

Abbreviations: CD, Crohn's disease; IBD, inflammatory bowel disease; SES‐CD, Simple Endoscopic Score for Crohn's Disease; UC, ulcerative colitis.

### 3.3 Effect of review

**Figure 1:**
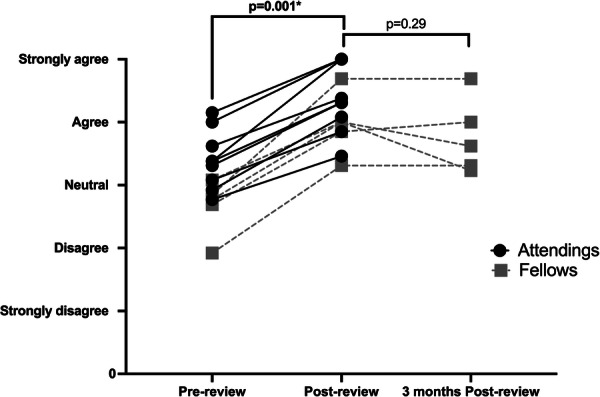
Attendings' and fellows' average question score (level of agreement of feeling comfortable with a topic) pre‐, post‐, and 3 months postreview of pediatric inflammatory bowel disease endoscopy. Attending' 3 months postreview was not evaluated. *p*‐value < 0.05 was considered significant.

All participants (*n* = 14) significantly increased their comfort pre‐ versus postreview on all 13 questions, as well as their average question score (Figure 1) and total score (*p* < 0.05 for all). This improvement was sustained in the fellows over 3 months without rereview, with no significant differences between post‐ versus 3 months post‐ for all questions, average question score (Figure 1), and total score (*p* > 0.05). Attendings' sustained improvement was not evaluated in this study.

### 3.4 Fellow and attending differences

Years in training/practice significantly strongly correlated with higher pre‐ (rho = 0.77) and post‐review (rho = 0.82) total scores (*p* < 0.01 for both). Significant differences between fellows' and attendings' median scores at baseline (prereview) included attendings being more comfortable identifying “normal” non‐IBD endoscopic findings (fellows 2, IQR 2–3 vs. attendings 4, IQR 4–4.5), identifying ileoscopy landmarks and findings (fellows 2, IQR 1.5–2 vs. attendings 4, IQR 3.5–4.5), having a higher average question score (fellows 2.8, IQR 2.3–3 vs. attendings 3.4, IQR 3–3.8) and a higher total score (fellows 36/65, IQR 30–39 vs. attendings 44/65, IQR 39–50) (*p* < 0.01 for all). These group differences were no longer significant postreview (*p* > 0.05).

## 4 DISCUSSION

This quality improvement initiative identified essential topics within pediatric IBD endoscopy where both fellows and attending pediatric gastroenterologists exhibited low levels of comfort. These areas improved significantly following review of recent guidelines, consensus/position papers, and standardized scores. The review successfully bridged the comfort level gap between fellow and attending physicians, and improvement among fellows was sustained for several months thereafter without further review. Knowledge retention is often difficult to achieve in pediatric GI training due to the sheer amount of new GI material fellows are expected to quickly master. Endoscopy provides a unique venue for “spaced education” as trainees can reinforce the learned endoscopy material with each endoscopy day that are often days to weeks apart. Spaced education has been found to significantly improve medical trainee's knowledge, and should continue to be explored in pediatric GI training.^7^

Education efforts should be targeted at postsurgical endoscopy (e.g., Rutgeert's score, and pouchoscopy), Crohn's endoscopic scoring (SES‐CD), IBD classification (Paris), and IBD dysplasia screening techniques. Postsurgical endoscopy is an important skill, as 50%–80% of CD patients and 10%–30% of UC patients will undergo at least one surgery in their lifetime,^16^ with a postsurgical colonoscopy in CD patients recommended 6–12 months postoperatively.^17^ Dysplasia screening is recommended to start 8–10 years after diagnosis in those with colonic IBD and every 1–2 years after diagnosis in IBD patients with primary sclerosing cholangitis (PSC).^10^ Although many patients will be in the adult age group once they meet this criteria, up to 5% of pediatric patients are diagnosed with PSC.^18^ In addition, the prevalence of very early onset‐IBD (<6 years old) is growing,^19^ resulting in an increased number of patients that will need dysplasia screening by a pediatric gastroenterologist before they transition to an adult gastroenterologist. Lastly, familiarity with the scoring and classification of IBD allows for consistency in patient care and decision making based on endoscopic findings (e.g., mucosal healing being defined as SES‐CD < 2 and Mayo = 0^20^). Pediatric gastroenterologists have previously demonstrated low agreement and concordance with adult gastroenterologists when scoring SES‐CD,^21^ underscoring the need for improvement in this area.

Our study's findings align with previously identified priorities by pediatric GI program directors, who emphasize the importance of endoscopic IBD knowledge in phenotypic classification of IBD (92% agree), landmarks/findings on pouchoscopy (61% agree), and classification of endoscopic severity of IBD using endoscopic indices (50% agree).^22^ While these topics were identified, this same study found that only a minority of fellows felt comfortable with these three areas (44%, 6%, and 19%, respectively).^22^ This further supports the need for continued endoscopy education even after fellowship.

The limitations of this study include being a single‐center study, incomplete participation of the entire GI division, as well as lack of available standardized objective assessment tool to assess endoscopy knowledge/comfort beyond self‐assessment. There is also a lack of validity in using comfort as an assessment tool, and further follow‐up studies should explore the assessment of confidence as a validated tool. There is a high likelihood of confirmation bias and placebo effect for the post‐review responses. The strengths of our study include using a feasible evidence‐based review that can be easily incorporated into ongoing education (i.e., GI division rounds) and highlighting actionable educational gaps among both trainees and practicing attendings. Furthermore, this pilot study had a positive impact in initiating a dialogue within our division on establishing pediatric endoscopy standards among providers. Future research should explore knowledge retention up to 12 months postreview session to further evaluate the longevity of the review education, as well as also evaluate attendings' long‐term knowledge retention.

## CONFLICT OF INTEREST STATEMENT

The authors declare no conflict of interest.

## Supporting information


**Appendix A.** Questions provided to fellows and attendings.
