# Supplementary material for: Self‐assessment with pediatric inflammatory bowel disease endoscopy scoring tools: A quality improvement pilot study
Source: JPGN Rep. 2025 Feb 6;6(2):132–6. doi: 10.1002/jpr3.70002 (PMC12078052; doi:10.1002/jpr3.70002)
Supplement: Supplementary file 1 — Appendix A. Questions provided to fellows and attendings. [file JPR3-6-132-s001.pdf]

Year of training: _______

Year graduated from Pediatric GI fellowship: ________

Strongly Disagree	Disagree	Neutral	Agree	Strongly Agree
1	2	3	4	5

Pre-session questions

1. I'm comfortable identifying IBD endoscopic findings: _______
2. I'm comfortable identifying incidental 'normal' non-IBD endoscopic findings: _______
3. I'm comfortable identifying IBD perianal findings: _______
4. I'm comfortable using the Mayo endoscopic score: _______
5. I'm comfortable using the SES-CD endoscopic score: _______
6. I'm comfortable using the Rutgeert postoperative endoscopic score: _______
7. I'm comfortable describing pouchoscopy landmarks and findings: _______
8. I'm comfortable describing ileoscopy landmarks and findings: _______
9. I'm comfortable using a bowel preparation rating scale (e.g. Boston Bowel Prep Scale): _______
10. I'm comfortable with techniques to improve the quality of the endoscopic image that is being taken: _______
11. I'm comfortable with endoscopic techniques for IBD dysplasia screening: _______
12. I'm comfortable classifying IBD with the Paris classification score for Crohn's and UC: _______
13. I'm aware of current recommendations on number/location of biopsies for suspected pediatric IBD: _______

Strongly Disagree	Disagree	Neutral	Agree	Strongly Agree
1	2	3	4	5

Post-session questions

1. I'm comfortable identifying IBD endoscopic findings: _______
2. I'm comfortable identifying incidental 'normal' non-IBD endoscopic findings: _______
3. I'm comfortable identifying IBD perianal findings: _______
4. I'm comfortable using the Mayo endoscopic score: _______
5. I'm comfortable using the SES-CD endoscopic score: _______
6. I'm comfortable using the Rutgeert postoperative endoscopic score: _______
7. I'm comfortable describing pouchoscopy landmarks and findings: _______
8. I'm comfortable describing ileoscopy landmarks and findings: _______
9. I'm comfortable using a bowel preparation rating scale (e.g. Boston Bowel Prep Scale): _______
10. I'm comfortable with techniques to improve the quality of the endoscopic image that is being taken: _______
11. I'm comfortable with endoscopic techniques for IBD dysplasia screening: _______
12. I'm comfortable classifying IBD with the Paris classification score for Crohn's and UC: _______
13. I'm aware of current recommendations on number/location of biopsies for suspected pediatric IBD: _______
